# Obese Adolescent with Factor V Leiden-Related Pulmonary Embolism: Case-Based Insight into Paediatric Thrombosis Management

**DOI:** 10.3390/diagnostics16172842

**Published:** 2026-09-03

**Authors:** Filip Bossowski, Magdalena Bossowska, Katarzyna Masłowska, Paweł Śliwko, Helena Żórawska, Kornel Semeran, Jacek Robert Janica, Artur Bossowski

**Affiliations:** 1 Department of Pediatrics, Endocrinology, Diabetology with Cardiology Divisions, Medical University of Białystok, ul. Jana Kilińskiego 1, 15-089 Białystok, Poland; 2 Department of Paediatric Radiology, Medical University of Białystok, ul. Jana Kilińskiego 1, 15-089 Białystok, Poland

**Keywords:** pulmonary embolism, venous thromboembolism, Factor V Leiden, inherited thrombophilia, deep vein thrombosis, anticoagulation, paediatrics, case report

## Abstract

**Background/Objectives:** Paediatric pulmonary embolism (PE) is rare but potentially lethal, and its diagnosis is complicated by non-specific presentations and multiple predisposing risk factors. We describe a 15-year-old girl who presented with features of pulmonary hypertension and was found to have extensive pulmonary embolism with right heart strain. **Methods:** The diagnosis was established with echocardiography, computed tomography pulmonary angiography (CTPA), and Doppler ultrasonography. **Results:** Her predisposing risk factors comprised obesity (BMI 33 kg/m^2^), a family history of thrombosis, and heterozygous Factor V Leiden. In retrospect, a six-week illness treated as bronchitis, with haemoptysis and exertional syncope, was the probable index embolic event; the markedly elevated right-sided pressures tolerated without haemodynamic collapse indicate a right ventricle that had adapted over that interval. Anticoagulation with low-molecular-weight heparin, titrated to anti-factor Xa activity, was followed by a vitamin K antagonist while antiphospholipid syndrome was excluded and then by rivaroxaban, with clinical, biochemical, and radiographic improvement. **Conclusions:** This report underscores the need for greater awareness of PE in children, the value of revisiting a recent respiratory illness as a possible index embolic event, and the evolving role of direct oral anticoagulants in its management.

## 1. Introduction

Pulmonary embolism (PE) is a clinical condition that can be life-threatening. It is caused by the sudden complete or partial blockage of the pulmonary artery or its branches by a thrombus that has migrated from the veins of the lower extremities or pelvis [1]. This blockage is a complication of venous thromboembolism (VTE) [2]. The blockage impairs blood flow in the pulmonary circulation, increases pressure, and places stress on the right ventricle [1]. In severe cases, this can lead to acute cardiac and respiratory failure or cardiac arrest [2].

Pulmonary embolism (PE), although a well-recognised condition in adults, is relatively rare in children. The incidence is between 0.14 and 0.9 per 100,000 children in the general paediatric population and 8.6 to 57 per 100,000 in hospitalised children. This incidence is increasing, possibly due to advancements in diagnostic techniques, the greater use of central venous catheters, improved survival rates in critically ill children, and a heightened awareness of the disease [2,3]. Unlike adults, in whom approximately 30% of PE cases are idiopathic, paediatric PE is predominantly associated with identifiable risk factors [4]. These risk factors can include: central venous catheters [5,6], infections [2], cancer, congenital heart disease [7], and inherited thrombophilias [2]. Children may also exhibit unique clinical features of PE, including silent or non-specific presentations, which can complicate diagnosis [8]. These presentations differ from the classic symptoms seen in adults, such as dyspnoea and pleuritic chest pain [2]. Another unique feature of paediatric PE is that it more commonly involves upper extremity DVT, which is frequently due to central venous line use [4]. This differs from adults, who predominantly experience lower extremity DVT [2].

D-dimer levels are elevated in most children (87%) at the time of presentation with PE [3]. Thrombophilia is not uncommon in children presenting with PE, particularly in adolescents and in cases that are not related to a central venous catheter [3]. The mortality rate associated with PE in children is significant (8.9%), and recurrence is a concern (12.5% recurrence rate) [3]. Anticoagulation therapy is the cornerstone of treatment for PE in children, and it results in complete or partial resolution in 82% of cases. However, anticoagulation therapy carries a substantial risk of haemorrhagic complications, which are observed in 21% of treated children. Long-term anticoagulation is often warranted when there is no acute, reversible, or transient risk factor [3]. For children with a history of PE in the context of risk factors, prompt re-initiation of anticoagulation is recommended if those risk factors return.

Unlike adults, paediatric patients often experience unique clinical features and risk factor profiles, which complicate early detection and highlight the importance of heightened clinical vigilance in at-risk populations.

## 2. Case Presentation

A 15-year-old girl was transferred from a regional hospital with suspected pulmonary hypertension. Approximately six weeks before admission, she experienced an episode of bronchitis characterised by high fever, dyspnoea, and haemoptysis. During that illness, she fainted twice upon standing and subsequently noted a significant decline in her exercise tolerance.

Three days before admission, her condition deteriorated, presenting with severe weakness, profound fatigue, worsening dyspnoea, and sharp, stabbing chest pain. Initial evaluations at the regional hospital revealed elevated N-terminal pro-brain natriuretic peptide (NT-proBNP) levels at 4195 pg/mL and significant tricuspid regurgitation on echocardiography, showing a maximal regurgitant velocity of 4.2 m/s and a pressure gradient of 70 mmHg. She was transferred to the University Children’s Clinical Hospital in Białystok for further evaluation and management.

Before the bronchitis episode, the patient had no history of cardiovascular symptoms and maintained an average level of physical activity. She was being treated with sertraline 100 mg daily for social anxiety and had no known allergies or other medications. Family history was notable for type 2 diabetes mellitus and a maternal history of lower extremity deep vein thrombosis (DVT).

On admission, the patient was alert, afebrile, and haemodynamically stable. Vital signs included blood pressure of 128/94 mmHg (measured with an appropriately sized large-adult cuff), heart rate of 115 beats/min (sinus tachycardia), respiratory rate of 18 breaths/min, and oxygen saturation (SpO_2_) of 96–97% on room air. Minimal physical activity led to a decrease in oxygen saturation to 90–93%, accompanied by tachypnoea and increased heart rate.

Physical examination revealed acne lesions on her chest and face. A dilated superficial vein was observed on her left calf. The patient was obese (weight 89 kg, BMI 33 kg/m^2^) with mild oedema around the ankles. Cardiac auscultation revealed a loud systolic murmur, graded 4/6 on the Levine scale, audible across the precordium, and an accentuated second heart sound at the base.

Laboratory tests showed elevated D-dimer levels at 2966 ng/mL, increased C-reactive protein (CRP) at 23 mg/L, elevated uric acid at 7.4 mg/dL, and NT-proBNP at 4195 pg/mL. Troponin levels remained negative throughout hospitalisation.

An electrocardiogram revealed right-axis deviation and sinus tachycardia at 115 beats per minute, with a PR interval of 0.16 s and a corrected QT interval of 0.42 s. Flattened T-waves were observed in the limb leads and leads V5–V6, along with negative T-waves from V1 to V4, indicating signs of right ventricular hypertrophy [9].

A transthoracic echocardiogram revealed marked right ventricular (RV) enlargement accompanied by signs of pressure overload, notably paradoxical interventricular septal motion. The right ventricular internal diameter in diastole measured 3.76 cm, and the right ventricular to left ventricular diameter ratio on computed tomography was 1.2, consistent with right ventricular dilatation and pressure overload. The right atrium (RA) was significantly dilated, measuring 44 × 50 mm, with an area of 19.5 cm^2^ (standard reference < 18 cm^2^). Severe tricuspid regurgitation (Grade IV) was present, characterised by a peak regurgitant jet velocity of 4.2 m/s and a peak tricuspid regurgitant pressure gradient of 71 mmHg, which highlighted the marked elevation of right-sided pressures.

**Figure 1:**
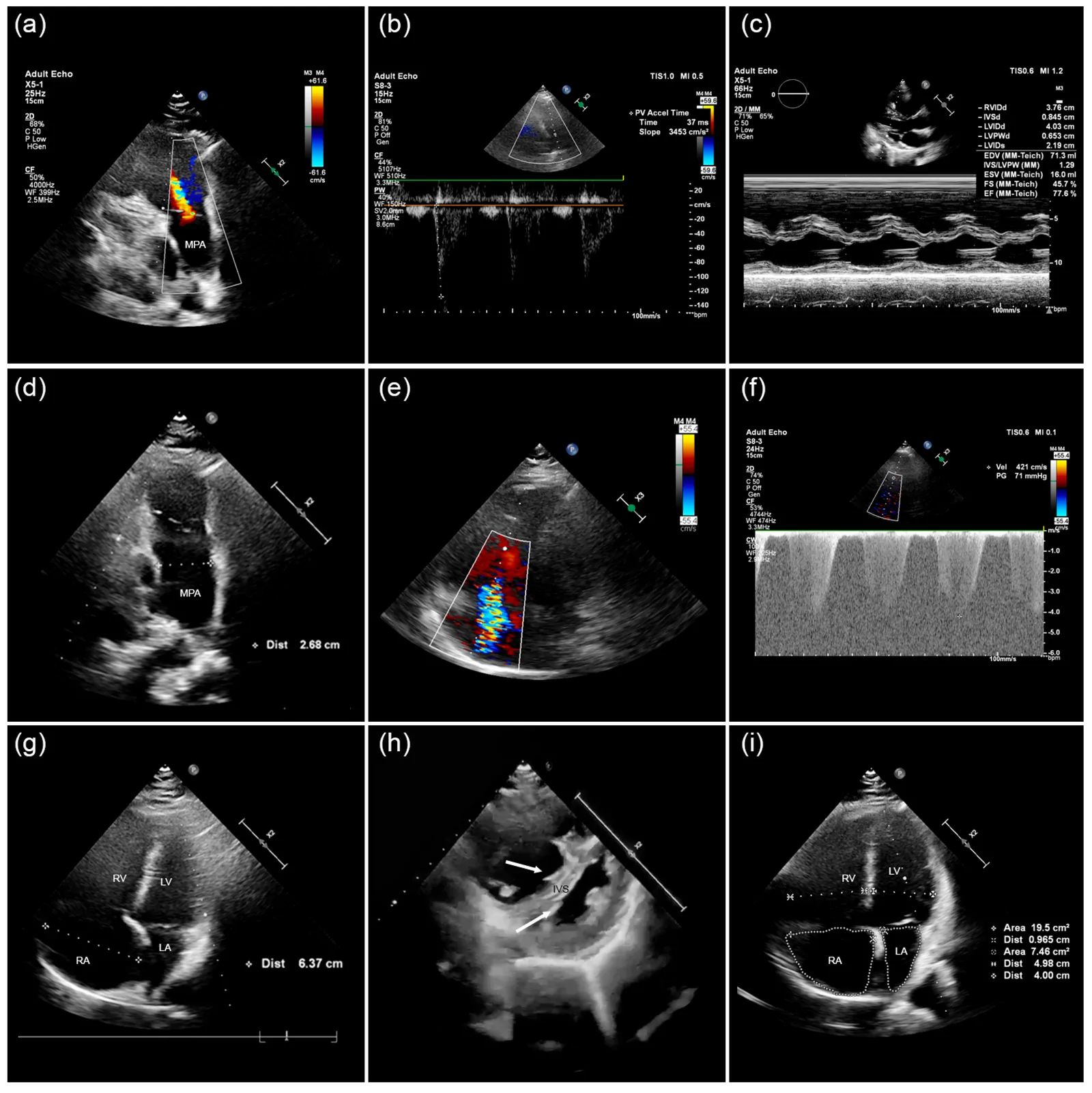
Composite echocardiographic images (**a**–**i**). (**a**) Parasternal short-axis view: grade II pulmonary regurgitation; (**b**) shortened acceleration time: pulmonary artery AcT of 37 ms; (**c**) parasternal long-axis view: prominent RV enlargement, septal bowing; (**d**) parasternal short-axis view: dilated main pulmonary artery (2.68 cm); (**e**) tricuspid regurgitation: pronounced regurgitant flow; (**f**) apical four-chamber view: elevated tricuspid inflow velocity (Vmax 4.2 m/s) and pulmonary artery pressure; (**g**) apical four-chamber view: marked right atrial enlargement; (**h**) flattening of the interventricular septum (arrows), giving the left ventricle a D-shaped configuration and indicating right ventricular pressure overload; (**i**) apical four-chamber view: enlarged right atrial area (19.5 cm^2^).

The main pulmonary artery (MPA) appeared notably dilated (2.68 cm in the parasternal short-axis view), while the right and left pulmonary arteries measured 17 mm and 18.5 mm, respectively. Pulmonary artery acceleration time (37 ms) was markedly reduced, and the additional PulmACT measurement (44 ms) further underscored elevated pulmonary pressures. Moderate (Grade II) pulmonary regurgitation was also observed, with a regurgitant gradient of 33 mmHg and a jet originating approximately 6 mm above the valve. A minimal mitral regurgitant signal was detected as well. Notably, near the MPA bifurcation into the right pulmonary artery a focal narrowing suggestive of thrombus was visualised on imaging (Figure 1).

**Figure 2:**
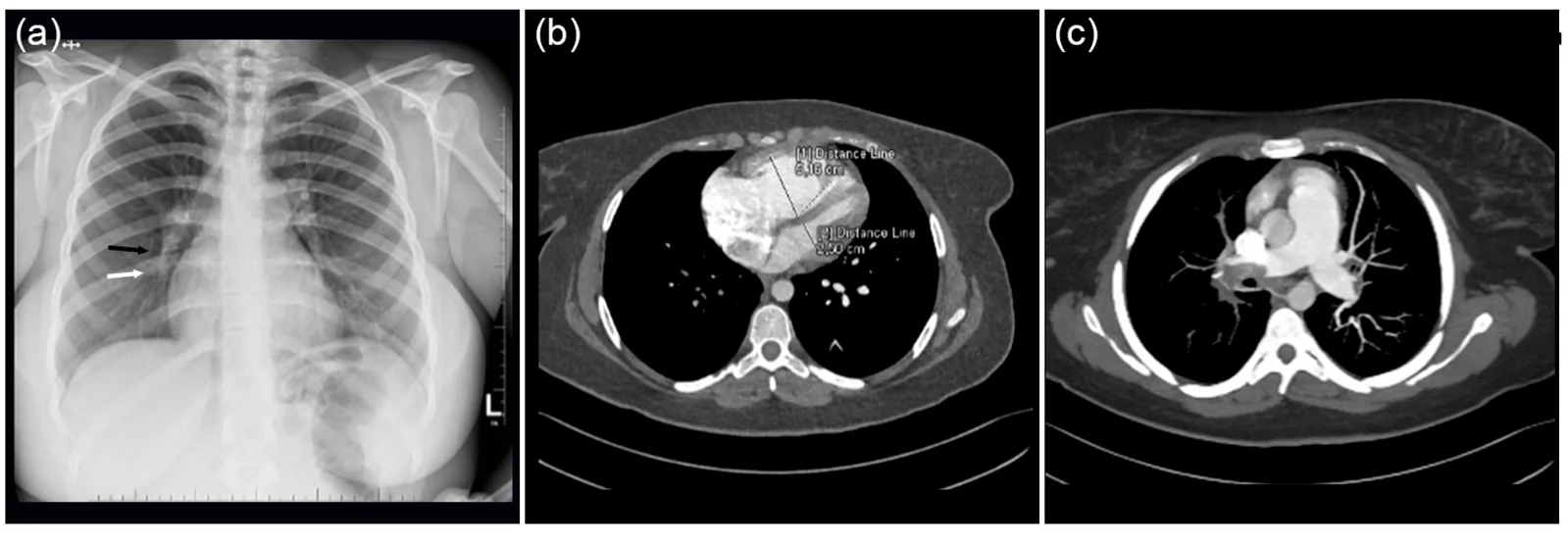
Chest radiograph and CT pulmonary angiography. (**a**) Posteroanterior chest radiograph showing an enlarged right descending (interlobar) pulmonary artery (black arrow; Fleischner’s sign) with an abrupt reduction in calibre distally (white arrow; Chang’s sign), both suggestive of pulmonary embolism. (**b**) Axial contrast-enhanced CT image demonstrating right ventricular enlargement, indicating right ventricular strain. (**c**) Axial contrast-enhanced CT image at the level of the pulmonary artery bifurcation, cranial to (**b**), showing filling defects in the pulmonary arteries, more extensive on the right.

Computed tomography pulmonary angiography revealed central and peripheral pulmonary emboli, more severe on the right side, with features suggestive of subacute thrombi and evidence of right ventricular dysfunction [10]. Thrombosis was also observed in the azygos vein. Additional findings included small ground-glass opacities and subpleural atelectatic consolidations in the lung fields (Figure 2).

**Figure 3:**
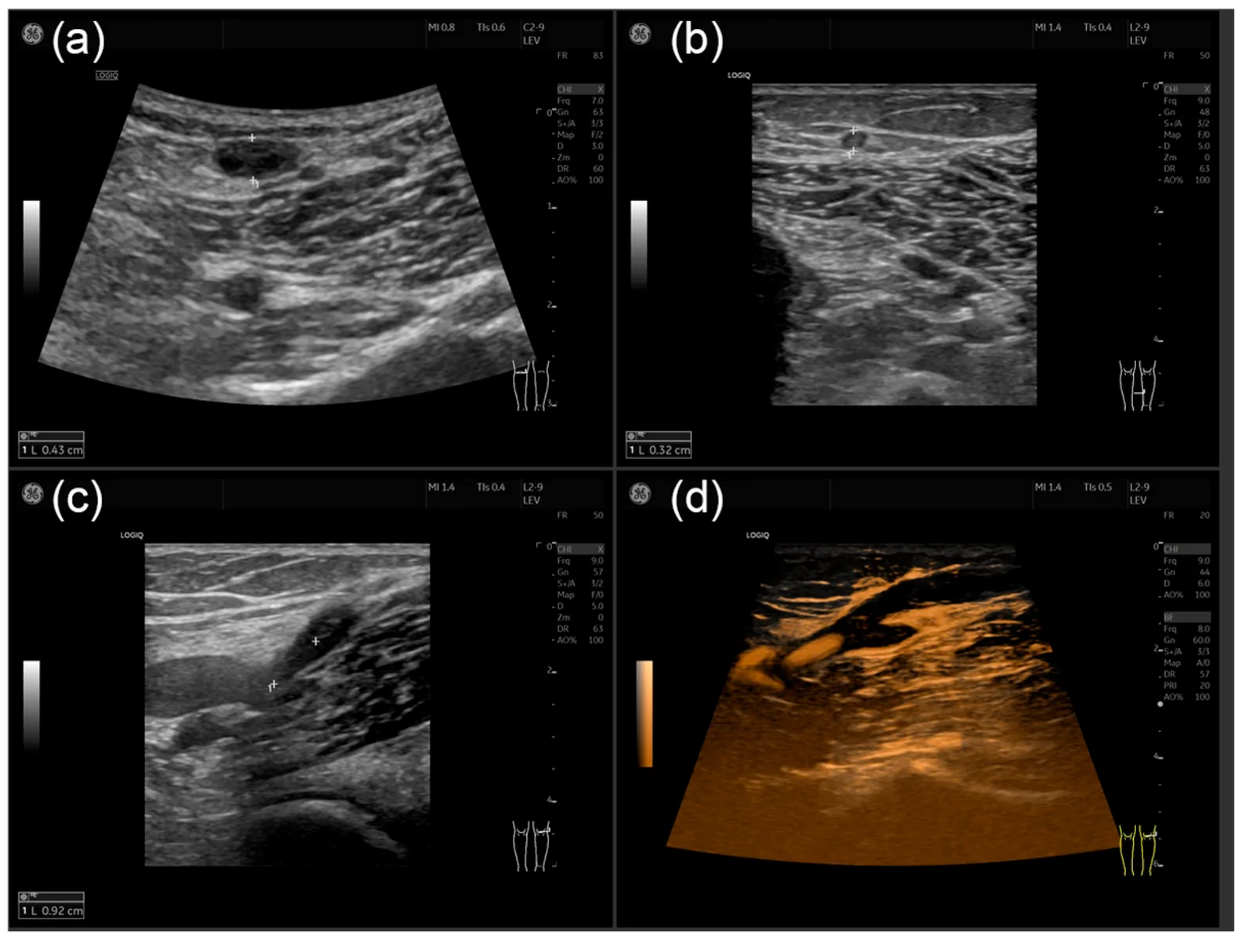
Doppler ultrasound of the left lower limb veins. (**a**) Transverse B-mode view showing an intraluminal echogenic focus within the great saphenous vein (measurement: 0.43 cm) (**b**) Transverse B-mode view demonstrating a similar echogenic region (0.32 cm) consistent with thrombus (**c**) Longitudinal section of the small saphenous vein (0.92 cm) illustrating mixed echogenicity, suggestive of clot in varying stages of organisation (**d**) B-flow GE longitudinal image depicting the thrombus within the superficial venous system, raising concern for possible proximal extension near the popliteal vein confluence.

Ultrasound examination of both lower limbs was performed, including full interrogation of the deep venous system. Superficial venous thrombosis was demonstrated in the left lower limb only, involving the great saphenous vein, the posterior arch vein, and the entire small saphenous vein up to the knee-joint level. The intraluminal thrombi measured approximately 3–4 mm in thickness and exhibited mixed echogenicity, suggesting varying stages of clot organisation. Of particular note, a rounded thrombus tip was visible at the small saphenous vein’s confluence with the popliteal vein, immediately adjacent to the saphenopopliteal junction, raising concern for potential extension into the deep venous system (Figure 3). Superficial thrombi lying close to the saphenopopliteal or saphenofemoral junction are regarded as at high risk of propagation and warrant therapeutic anticoagulation equivalent to that used for deep vein thrombosis. No thrombus was identified within the deep venous system of either limb.

A thrombophilia workup revealed normal protein C, protein S, homocysteine, and antithrombin III levels. Antiphospholipid antibodies were negative, including anti-cardiolipin antibodies (IgM and IgG), anti-beta-2 glycoprotein I antibodies (IgM and IgG), and lupus anticoagulant. Genetic testing identified a heterozygous Factor V Leiden mutation, while no prothrombin gene (G20210A) mutation was detected.

After consultation with paediatric haematology specialists, systemic thrombolysis with alteplase was deemed unnecessary due to her stable condition. Anticoagulation therapy with low-molecular-weight heparin (LMWH) was initiated at a dose of 1 mg/kg twice daily (enoxaparin 90 mg subcutaneously every 12 h), along with spironolactone, furosemide, and supplemental oxygen therapy. Due to elevated anti-factor Xa activity (1.4 IU/mL), the LMWH dose was reduced to 80 mg twice daily.

Twelve days later, acenocoumarol was added, overlapping with LMWH until a therapeutic International Normalized Ratio (INR) of 2–3 was achieved, after which LMWH was discontinued. Laboratory results showed a gradual decline in NT-proBNP and D-dimer levels.

During hospitalisation, the patient’s symptoms improved significantly, with reduced dyspnoea and resolution of tachycardia. She reported no further episodes of chest pain or hypotension. Repeat echocardiography demonstrated improved right ventricular function, increased pulmonary acceleration time to 74 ms, and reduced tricuspid regurgitation to grade II with a maximal systolic pressure gradient of 46 mmHg. Follow-up computed tomography pulmonary angiography showed slight improvement compared to the initial imaging, with persistent but reduced bilateral filling defects in the pulmonary arteries, consistent with chronic pulmonary embolism. Ultrasound of the lower limbs indicated recanalisation of the previously thrombosed veins and no evidence of new thrombus formation.

After excluding antiphospholipid syndrome, anticoagulation was transitioned to rivaroxaban 20 mg once daily, in accordance with the weight-tiered paediatric regimen established in the EINSTEIN-Jr trial for adolescents weighing 50 kg or more, which was well tolerated [11]. A minimum treatment duration of 12 months was planned, with the decision on extended anticoagulation to be based on repeat imaging, resolution of right ventricular strain, and her persistent unprovoked risk profile. She was discharged several weeks later with marked improvement in her general condition, normalised NT-proBNP levels, and improved echocardiographic findings. She was advised to continue anticoagulation therapy and scheduled for outpatient follow-up.

## 3. Discussion

### 3.1. Diagnostic Challenges

Diagnosing pulmonary embolism (PE) in paediatric patients is particularly challenging due to its rarity, overlapping clinical presentations, and the frequent presence of underlying conditions [8]. A 15-year-old girl presenting with dyspnoea, chest pain, syncope, and reduced exercise tolerance prompted an initial suspicion of pulmonary hypertension. This suspicion was reinforced by elevated NT-proBNP levels and echocardiographic findings of significant tricuspid regurgitation and right ventricular dysfunction. Such overlap between PE and pulmonary hypertension complicates prompt diagnosis. In this case the overlap was compounded by the timing of presentation. Six weeks before admission the patient had an illness treated as bronchitis, comprising fever, dyspnoea, haemoptysis, and two orthostatic syncopal episodes, followed by a sustained decline in exercise tolerance. Haemoptysis and syncope are not typical of uncomplicated bronchitis, and computed tomography pulmonary angiography subsequently demonstrated features of subacute rather than acute thrombus, with persistent filling defects on follow-up imaging. Taken together, these observations indicate that this episode was in all likelihood the index embolic event rather than a respiratory infection. The haemodynamic findings support the same conclusion. In an unconditioned right ventricle, acute pulmonary arterial occlusion generally produces acute right heart failure and haemodynamic collapse once systolic pressures exceed approximately 40 to 45 mmHg. This patient tolerated a tricuspid regurgitant gradient of 71 mmHg while remaining normotensive and haemodynamically stable on room air, which is only compatible with a right ventricle that had undergone compensatory hypertrophy and pressure adaptation over a period of weeks. Subacute pulmonary embolism in adolescents may therefore present with the physiological signature of established pulmonary hypertension, and a recent respiratory illness accompanied by haemoptysis or syncope should be revisited as a potential index embolic event. Electrocardiogram findings of right-axis deviation, right ventricular hypertrophy, and T-wave inversions, while indicative of right heart strain, were indeterminate. Diagnostic tools that are tailored for paediatric use are scarce; D-dimer assays and clinical probability scores are less reliable than in adults [12]. Here, the acute onset of symptoms and the patient’s risk factors raised clinical suspicion, prompting further investigation. Computed tomography pulmonary angiography (CTPA), the current imaging standard, confirmed PE by visualising central and peripheral emboli and an azygos vein thrombus. Although echocardiography showed right ventricular dysfunction, CTPA provided more definitive evidence of right-sided strain, including right ventricular enlargement relative to the left, thereby distinguishing PE from other conditions with similar presentations.

### 3.2. Role of Risk Factors

The patient’s identified risk factors—including obesity (BMI of 33 kg/m^2^), a family history of thrombosis, and a heterozygous Factor V Leiden mutation—collectively elevated her risk of venous thromboembolism (VTE) [13]. Factor V Leiden, the most common inherited thrombophilia, confers resistance to activated protein C, promoting a hypercoagulable state [14]. Obesity similarly contributes to VTE risk by creating a prothrombotic milieu and reducing mobility [15]. The interplay of these coexisting factors likely intensified the patient’s thrombotic susceptibility. The finding of azygos vein thrombosis merits separate comment since it is exceptionally rare in a paediatric patient without a central venous catheter. Two mechanisms appear plausible. Severe tricuspid regurgitation with a markedly dilated right atrium (19.5 cm^2^) would be expected to raise central venous pressure and generate retrograde venous stasis within the azygos system, favouring thrombus formation in situ. Alternatively, the appearance may represent thrombus reaching the azygos system through collateral venous pathways. The former seems the more likely explanation here, given the severity of right-sided pressure and volume overload documented on echocardiography, although the two are not mutually exclusive. It is also relevant that isolated superficial venous thrombosis is an uncommon sole source for a clot burden of this magnitude, which is why exclusion of deep, pelvic, iliac, and caval thrombosis was considered necessary in this patient [13].

### 3.3. Management Strategies

The primary aim of therapeutic intervention in paediatric PE is to arrest thrombus propagation and mitigate associated morbidity and mortality [16]. In this case, initial anticoagulation therapy with low-molecular-weight heparin (LMWH) was selected due to its favourable pharmacokinetics, ease of dosing, and established safety profile in children [17].

Enoxaparin dosing was titrated according to anti-factor Xa levels, ensuring effective anticoagulation while minimising bleeding risk.

The significant influence of developmental stage on drug metabolism and pharmacokinetics in paediatric populations requires an individualised approach to dosing of this low-molecular-weight heparin. Body habitus is an equally important determinant in this age group. Initial dosing at 1 mg/kg twice daily calculated on total body weight (enoxaparin 90 mg) produced a supratherapeutic anti-factor Xa level of 1.4 IU/mL, prompting dose reduction. Low-molecular-weight heparins are hydrophilic and distribute predominantly within the intravascular compartment rather than into adipose tissue, so dosing strictly by total body weight in obese adolescents systematically overestimates requirements and risks over-anticoagulation. Anti-factor Xa monitoring is therefore particularly valuable in this population, and clinicians should anticipate the need for downward adjustment rather than treating an initial supratherapeutic result as unexpected.

Although thrombolytic therapy can be considered in life-threatening PE or cases refractory to standard anticoagulation, it was not indicated here due to the patient’s haemodynamic stability and absence of massive PE [18]. Given the significant bleeding risk associated with thrombolysis, its use must be thoughtfully justified.

The patient was transitioned from LMWH to oral anticoagulation with acenocoumarol, overlapping until a therapeutic International Normalized Ratio (INR) was achieved. Maintaining stable INR levels proved difficult.

The need for frequent monitoring underscored the practical advantages of using rivaroxaban, a DOAC with fixed dosing that could potentially eliminate the need for periodic testing [19]. Recent studies support the safety and efficacy of rivaroxaban in paediatric venous thromboembolism (VTE), highlighting its potential as a viable long-term anticoagulation strategy. The sequence of anticoagulants used here was deliberate rather than incidental. A vitamin K antagonist was maintained until antiphospholipid syndrome had been formally excluded, because direct oral anticoagulants are less effective than vitamin K antagonists in high-risk antiphospholipid syndrome, and only then was rivaroxaban introduced. In a patient with confirmed inherited thrombophilia and a strong family history, this ordering avoids committing a young patient to a direct oral anticoagulant before an antibody-mediated cause has been ruled out. One further interaction warranted attention: the patient was taking sertraline 100 mg daily, and selective serotonin reuptake inhibitors deplete intra-platelet serotonin and may impair primary haemostasis. The combination with potent anticoagulation, particularly during periods of elevated anti-factor Xa activity, represents an additive bleeding risk that should be recognised and monitored rather than assumed to be negligible [20].

### 3.4. Monitoring and Dose Adjustment

Anti-factor Xa levels, maintained at 0.5–1.0 U/mL four to six hours post-injection, guided LMWH dosing adjustments, as aPTT is prone to be an unreliable measure for LMWH therapy [21]. Serial NT-proBNP and D-dimer measurements assessed right ventricular strain and thrombus resolution [22]. Repeated echocardiography and computed tomography pulmonary angiography (CTPA) further evaluated changes in clot burden and improvements in cardiac function. Several limitations should be acknowledged. As a single case, these observations are illustrative rather than generalisable. The identification of the six-week prodrome as the index embolic event is retrospective and inferential, since no imaging was obtained at that time. Quantitative indices of right ventricular systolic function were not recorded systematically at every timepoint, which limits objective comparison of recovery. Finally, chronic thromboembolic pulmonary hypertension has not been formally excluded, and the follow-up required to do so is ongoing.

### 3.5. Long-Term Management and Follow-Up

Given the finding of the patient’s inherited thrombophilia and multiple risk factors, long-term anticoagulation therapy was initiated, with the duration to be periodically reassessed according to clinical guidelines and evolving risk profiles [22].

The patient received guidance on adopting lifestyle changes, including structured weight management and a gradual increase in physical activity, to attenuate the risk of future thrombotic events and cardiovascular complications. The patient and her family were educated on recognising potential signs of VTE recurrence—such as leg pain, swelling, chest discomfort, or new-onset shortness of breath—to ensure timely medical assessment.

Regular follow-up appointments were scheduled to monitor the effectiveness and safety of anticoagulation, evaluate potential complications, and reinforce adherence to lifestyle adjustments. Coordinated care involving paediatric cardiology (to assess cardiac function and potential pulmonary hypertension), haematology (to manage anticoagulation and monitor for complications), and psychological support services (to address mental health and treatment adherence) ensured a comprehensive, multidisciplinary approach to long-term management and improved quality of life.

## 4. Conclusions

The principal lesson of this case is diagnostic. In an adolescent presenting with features of pulmonary hypertension, subacute pulmonary embolism should be considered before the picture is attributed to primary pulmonary vascular disease, particularly where a recent respiratory illness was accompanied by haemoptysis or syncope. Here a six-week episode treated as bronchitis was, in retrospect, the probable index embolic event, and the tolerance of markedly elevated right-sided pressures without haemodynamic collapse indicated a right ventricle that had adapted over weeks rather than hours. This case demonstrates that, though rare in children, PE can be managed using strategies adapted from adult practice, including DOACs such as rivaroxaban. While paediatric-specific data remain limited, this outcome adds to the emerging evidence supporting DOAC use in younger patients.

Timely diagnosis, recognition of underlying factors, and appropriate therapy were associated with marked clinical, biochemical, and radiographic improvement. Chronic thromboembolic pulmonary hypertension cannot be considered excluded on the basis of the present findings, since perfusion defects persisted on follow-up imaging; formal assessment requires ventilation–perfusion scanning and right heart catheterisation after at least three months of anticoagulation, and this is planned as part of her continuing follow-up. Still, further paediatric-focused research and guidelines are necessary to refine diagnostic approaches, especially for adolescents with inherited thrombophilias, establish optimal dosing, and improve long-term outcomes in this unique patient population.

## Data Availability

The original contributions presented in this study are included in the article. Further inquiries can be directed to the corresponding author.
